# Signal Pathway of Estrogen and Estrogen Receptor in the Development of Thyroid Cancer

**DOI:** 10.3389/fonc.2021.593479

**Published:** 2021-04-28

**Authors:** Jian Liu, Tianmin Xu, Li Ma, Weiqin Chang

**Affiliations:** ^1^Department of Gynaecology and Obstetrics, Jilin University Second Hospital, Changchun, China; ^2^Department of Thyroid Surgery, Jilin University Second Hospital, Changchun, China

**Keywords:** thyroid cancer, estrogen, estrogen receptor, signal pathway, thyroid cancer cells

## Abstract

The molecular mechanisms underlying the development of thyroid cancer (TC) have been examined through extensive experiments. A large number of studies have shown that the incidences of thyroid cancer in women is much higher than that in men, so estrogen plays a key role in the development of thyroid cancer. Estrogen plays its growth-promoting role through classical genomic and non-genomic pathways mediated by membrane-bound estrogen receptors. It also can affect tumor progression by regulating the tumor microenvironment. We summarize the understanding of molecular mechanisms of estrogen signaling pathways in thyroid cancer. Furthermore, it will provide a new target for the treatment of thyroid carcinoma by blocking estrogen and its related action pathway.

## Introduction

Although endogenous estrogen has normal and beneficial physiological effects, the levels of estrogen changed abnormally relating to the development of some kinds of cancer. The main intracellular estrogen is 17-β estrogen (E2). Estrogen performs its biological role in target sites mainly through combining with estrogen receptor (ER) that is an intracellular receptor including ERα and ERβ. A large number of studies have been developed to explore the biological mechanisms associated with estrogen and ER. Estrogen and ER binding to form complexes acting as ligands to activate transcription factors is a classical mode of action, which can regulate the expression of target genes. For example, cathepsin D and cyclinD1 are identified as endogenous E2 target genes, and their transcription is promoted by estrogen in an ER-dependent manner. Another way is that estrogen produces rapid signal transduction via binding to membrane-associated ER (mER). This non-genomic action is different from gene transcription, which is generated from outside the nucleus so it is very quick (1).

There are significant sex differences in the case rate of thyroid cancer, and a large number of epidemiological and experimental results have shown that the growth and development of TC involves the effects of female sex hormones, especially E2. Hormone age dependence and sex-specific factors may be responsible for this. The sharp increase in morbidity in females occurred around the age of 20, peaked near menopause at the age of 50 years, while peaked in men around the age of 70 years. Estrogen and ER mediated are most likely to cause differences in thyroid cancer between men and women. However, specific mechanism occurrence has not been identified (2). Estrogen is an effective driver for the growth of benign or malignant thyroid cells, and E2 helps to understand new mechanisms of thyroid cancer cell proliferation and population growth. Moreover, ER expression is related with TNM staging and peritumoral inflammatory infiltration in thyroid cancer (3, 4).

ERα and ERβ are both expressed in TC cells, and the biological function of estrogen is regulated by these two related but different estrogen receptors at target tissues (5). E2 treatment facilitates ERα expression rather than ERβ. Researches have proven that the expression level of ERα is increased, but the expression level of ERβ is reduced or even absent in thyroid cancer cells. ERα agonists can increase the proliferation of TC cells, while enhanced expression of ERβ or the use of ERβ agonists can decrease the proliferation of TC cells (6). ERα marker index or percentage of ERα-positive cells in TC was significantly higher than that in normal thyroid glands (7). The imbalance between increased ERα and decreased ERβ was found in multiple kinds of TC cells, such as thyroid medullary cancer cell lines (TT), anaplastic thyroid cancer (ACT) cell lines (ARO), and papillary thyroid carcinoma (PTC) cell lines (KAT5), which may alter cell behavior (8, 9). PES1 is a BRCT domain-containing protein that upregulates ERα/ERβ protein proportion and promotes proliferation of PTC cells (10). These results suggest that estrogen may affect the development, physiology, and pathology of the human thyroid, and that these effects become more pronounced in tumors through ERα, especially in premenopausal women (7). Researches also indicated that the increase in ERα expression in postmenopausal PTC cells is more pronounced and is associated with PTC invasion (11). Overall, the influence of estrogen on TC cell growth depends on the balance between ERα and ERβ.

Estrogen has a variety of genetic and epigenetic changes in the occurrence and development of thyroid cancer, in which activation of phosphatidylinositol-3 kinase (PI3K)/AKT signal pathway and mitogen-activated protein kinases (MAPK) signal pathway due to mutations are critical in cancer progression. This review summarizes the pathways involved in the molecular mechanisms of estrogen and ER in thyroid cancer, and in the future, we can select suitable targets for treatment to improve the prognosis of TC patients.

## Molecular mechanism of Estrogen Receptor-Mediated

Thyroid cancer is a class of endocrine malignancy, and a lot of insights have been gained on the molecular pathogenesis (12). Chromosomal rearrangements of RET/PTC, and PAX8/PPARγ (peroxisome proliferator-activated receptor gamma) and point mutations in BRAF and RAS genes are the most common in thyroid cancer (13). There are also many studies on the effects of estrogen and its receptors in its pathogenesis. E2 can induce cell proliferation, adhesion, and migration through ER expressed in many thyroid cancer cells. E2 induced the migration of PTC cell line BCPAP, which is mediated at least in part by increased vimentin and MMP-9 expression and decreased E-cadherin expression (5). Downregulation of tumor suppressor protein β-catenin in E2-treated Nthy-ori 3-1 and BCPAP cell lines is also associated with tumor invasion whether *in vitro* or *in vivo* (14). E2 positively regulates HER2 expression by increasing steroid receptor co-activator (SRC)-1 and cyclinD1 protein expression in follicular thyroid cancer (FTC)-133 cell lines, and these proteins are related with poorly differentiated tumor and disease recurrence (15). E2 can also promote PTC cell proliferation by ERα/specificity protein-1 (Spl)-mediated upregulation of heat shock protein 27 (Hsp27), while ERβ inhibits this pathway, and ERα levels are more than twice as high as ERβ. ERα/SP1 upregulates Hsp27 by interacting with procaspase-3, which promotes cell proliferation and resistance to apoptosis (16).

Other studies have shown that ERβ enhances the expression of some apoptosis-inducing factors and pro-apoptotic molecules caspase-3 through interaction with PPARγ, thereby significantly improving the cell proliferation and migration in TC. Overexpression of ERα or ERβ decreased PPARγ protein activity, while the use of diarylpropiolnitrile (DPN), a kind of ERα and ERβ agonist, increased the expression of PPARγ protein. Giving rosiglitazone (RTZ), a PPARγ ligand, counteracted ER effects while reducing ER expression. Functionally, ERα activation counteracts PPARγ inhibition of cell function, but ERβ activation aggregates it and induces apoptosis (17). In another study, the expression of ERβ was similarly elevated in the context of higher ERα levels, which was associated with the expression and content of neurogenic factor BRN-3α and TRIM16 (a nuclear protein that regulates estrogen receptors). BRN-3α is closely related to ER receptors, and the content of TRIM16 protein is related to the expression of ERβ. TRIM16 is characterized by “anti-estrogen” activity, and its anti-estrogen action is mainly concentrated on ERβ, but the inhibitory effect of this receptor in the TC has not been confirmed, probably due to the biological characteristics of the tumor (18).

## Estrogen and Estrogen Receptor-Mediated Pathways in Thyroid Cancer Cells

**Figure 1:**
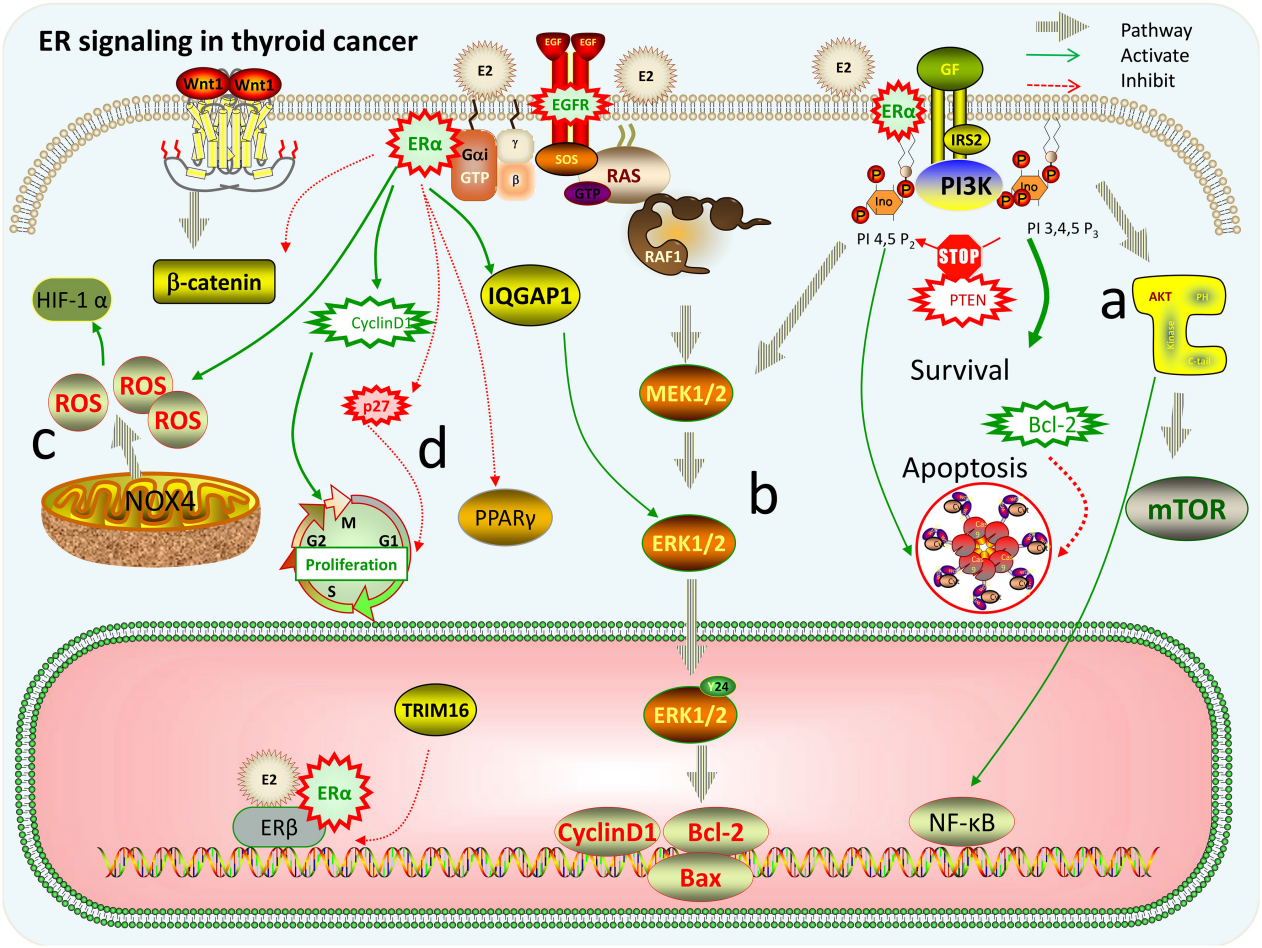
Estrogen and estrogen receptor-mediated pathways in thyroid cancer. **(a)** PI3K/AKT/mTOR pathway: activation of PI3K promotes cell proliferation and differentiation. PTEN gene can prevent PI3K phosphorylation and promote apoptosis, while activation of Bcl-2 gene promotes cell proliferation; AKT can promote NF-κB nuclear translocation, activate target genes, and promote cell survival. **(b)** Ras/Raf/MEK/extracellular signal-regulated kinase (ERK) pathway: E2 can make mitogen-activated protein (MAP) kinase isoenzymes and ERK 1/2 strongly phosphorylated, and then promote the expression changes of apoptosis-related factors, such as Bcl-2, Bax, and cyclinD1, increasing the ability of cell proliferation and survival. ERα can interact directly with IQGAP1 to phosphorylate ERK1/2 thereby promoting cell proliferation. **(c)** Reactive oxygen species (ROS)-related pathways: E2 can stimulate ROS production by acting on NOX4 that may be located in the plasma membrane, endoplasmic reticulum, nuclear membrane, and mitochondria, and then ROS induces the overexpression of HIF-1α. **(d)** E2 increases cyclin D1 protein expression and reduces the expression of p27 and β-catenin by ERα. Overexpression of ERα or ERβ decreased the activity of PPARγ protein, thereby significantly promoting the proliferation and migration of thyroid cancer cells. TRIM16 has “anti-estrogen” activity, and its anti-estrogen action is mainly concentrated in ERβ.

Through the membrane-bound estrogen receptor, E2-mediated genomic transcriptional classic pathway that occurs in the nucleus and non-genomic transcriptional pathway that occurs outside the nucleus develop their proliferative function in thyroid cancer cells (19). It is known that the non-genomic role of E2 has an important function in thyroid tumorigenesis mainly through activating PI3K/AKT and extracellular signal-regulated kinase (ERK) 1/2 signaling pathways (1). ERs are associated with tyrosine kinase signaling pathway MAPK and PI3K. ICI (an ER antagonist) as well as inhibitors that inhibit E2-activated PI3K and MAPK pathway by a non-genomic action can prevent cell proliferation induced by E2. The MEK/ERK pathway and PI3K/AKT pathways are two recognized signaling pathways that regulate autophagy. The AKT/mTOR pathway affects the regulation of autophagy, while the MEK/ERK pathway promotes the regulation of autophagy (20). There is growing evidence that E2 activates MEK/ERK pathways in ERα-positive cells, for example, E2 stimulates ERK1/2 pathway in thyroid cancer cell lines (KAT5 and WRO) through ER (21). Estrogen and estrogen receptor-mediated pathways in thyroid cancer are shown in Figure 1. In TC, the signaling pathway related to estrogen for *in vitro* treatment is mentioned in Table 1, and the effect of estrogen in mice with thyroid cancer is evaluated in Table 2. High levels of estrogen in TC may stimulate these pathways through the chromosomal rearrangements of tyrosine receptor kinases TRKA, leading to RET/PTC or BRAF gene mutations (20). According to these molecular mechanisms of pathways, many new treatments can be used for different types of thyroid cancer (Table 3).

**Table 1:** The signaling pathway of estrogen *in vitro*.

**Cell lines**	**Pathway**	**Function**	**Reference**
BHP10-3 cell lines (PTC)	AKT/mTOR signaling	E2 enhanced the expression of ERα/ERβ and GPR30 and could quickly phosphorylate AKT/mTOR.	(22)
WRO cell lines(FTC)and FRO cell lines(ATC)	GPER/ERK&AKT/NF-κB signaling pathway	Cd-induced proliferation, invasion, and migration of GPER-positive thyroid cancer cells.	(23)
FTC-133(FTC),Nthy-ori 3-1 cell lines (normal thyroid follicular epithelial cells) and simian-derived COS-7 cell lines	ERK pathway	IQGAP1 knockdown represses cell proliferation and invasion, and ERα transcriptional activity.	(24)
NPA87,KAT5 cell lines (PTC) and WRO, TPC1 cell lines (FTC)	ERK pathway	E2 increased thyroid cancer cells proliferation.	(21)
KAT5 cell lines (PTC), FRO cell lines (FTC) and ARO cell lines (ATC)	ERK1/2-related pathway	E2 promoted thyroid cancer cells proliferation.	(8)
BCPAP cell lines (PTC) and ML-1 cell lines (FTC)	VEGF signaling pathways	E2 induces cells to secrete paracrine factors that lead to endothelial cell tubulogenesis.	(25)
BHT-101 and CAL-62 cell lines (ATC)	MAP kinase signaling	Treatment with GSK5182 (an inverse agonist of ERRγ) resulted in dose- and time-dependent increases in iodide uptake in cells.	(26)
HTC-TSHr,XTC 133 cell lines	MAP kinase pathway ROS and	E2 increased cell proliferation and cyclinD1 protein expression levels	(27)
Nthy-ori 3-1 cell lines (human normal thyroid cell),BCPAP and BCPAP-ER cell lines (PTC)	ERK1/2-related pathways	ER contributes cell proliferation, and enhances autophagy.	(28)
TPC-1 and K-1 cell lines (TC) and 293 T cells	lncRNA-H19/miRNA-3126-5p/ ERβ	H19/miR3125-5p regulates stem-like properties upon E2 treatment through ERβ in PTC cells.	(29)
CD4 + T cells from PTC patients	miR-103/GPER1	SMI inhibited the differentiation of CD4 + T cells into Treg cells	(30)
K1,W3 cell lines (PTC)and Nthy-ori 3–1 cell line (FTC)	miR-219-5p/ ERα	miR-219-5p suppressed cell proliferation and migration, and promoted apoptosis	(31)

**Table 2:** The effect of the signaling pathway of estrogen *in vivo*.

**Model**	**Treatment**	**Function**	**Reference**
Male C57BL/6 mice (6–8 weeks old)	Hyperthyroidism was induced by injecting TH	The livers from TH-treated mice had significantly more mitochondria in an ESRRA-dependent manner.	(32)
BALB/c nude female mice (4–6 weeks old)	K-1 (NTC and shERβ) cells were subcutaneously injected	The tumor volumes in shERβ-1 and shERβ-2 groups were apparently smaller than NTC group	(29)
PTEN mutant mice	Ovariectomy was performed on 4-week-old mice	Complete estrogen ablation reduced the proliferative index of female thyroids to the same levels observed in mutant males	(33)

**Table 3:** Novel therapies for different types of thyroid cancer.

**Type of thyroid cancer**	**Molecular mechanism**	**Therapy**	**Reference**
Papillary thyroid cancer	ERα was positively correlated with Ki-67, while ERβ1 was negatively correlated with mutant P53.	Therapeutic approaches to PTC with ERα-specific antagonists or ERβ1-specific agonists.	(4)
Papillary thyroid carcinoma	Bisphenol A (BPA) or 17-β estrogen (E2) could quickly phosphorylate AKT/mTOR.	ICI and G-15 may have the potential to be used as anti-thyroid cancer agents.	(22)
Papillary thyroid cancer and anaplastic thyroid cancer	The enhancement of ERβ by its agonists or the activation of PPARγ by its ligands induces apoptosis.	The cross-talk between ER and PPARγ can provide a new therapeutic strategy against thyroid cancer.	(17)
Follicular thyroid cancer	Estrogens can activate PI3K pathway and control p27 levels through Skp2.	Circulating estrogens are directly responsible for the increased female susceptibility.	(33)
Anaplastic thyroid cancer	ERRγ inverses agonists enhances the NIS-mediated radioiodine uptake in cells with either KRAS or BRAF mutation	Discovery of ERRγ inverse agonists to facilitate radioiodine therapy *in vitro*.	(34)
Papillary thyroid cancer and follicular thyroid cancer	Estrogen can induce a proangiogenic endothelial cell phenotype through ER and VEGF signaling.	The effect of antiestrogenic therapy targeting tumor angiogenesis can be enhanced through VEGF inhibition.	(11)
Papillary thyroid cancer.	Autophagy induced by estrogen/ERα is associated with generation of ROS, activation of ERK1/2.	Drugs that inhibit autophagy are available for use in the clinic, including CQ and its derivative hydroxychloroquine.	(28)
Papillary thyroid cancer and follicular thyroid cancer	Estrogen contributes to angiogenesis of estrogen responsive thyroid cancer.	Fulvestrant and DIM, inhibit VEGF secretion, which can be used as a part of therapeutic regimen	(35)

### PI3K/AKT Pathway

PI3K is a core diver in the signaling cascade of thyroid hyperplasia and neoplastic diseases, and the constitutive activation of PI3K gives the thyroid follicular cell proliferative advantage (36, 37). These include PTEN mutations, PIK3CA mutations, RAS mutations, and increased amplification, and copy of AKT, PIK3CB, PDK1, and multiple kinase-related genes. These gene mutations are very common in follicular thyroid cancer, and some of them are more common for ACT that is a kind of rare thyroid cancer with poor prognosis. The PTEN epigenetic silencing is a major event in negatively regulating PI3K/AKT pathway and is bound up with genetic alterations that activate PI3K/AKT pathways, which constitutes a unique enhancing mechanism (38). Using PI3K/AKT pathway inhibitors, LY294002 can significantly inhibit autophagy in PTC cells.

While PI3K pathways have a crucial function in balancing homeostasis in thyroid hyperplasia, FTC occurring in thyroid-specific PTEN –/– models demonstrate that constitutive PI3K signaling activation is not sufficient for tumor transformation, and that a synergistic effect with PTEN and KRAS oncogenic allele deletion is required to lead to rapid development of thyroid follicular carcinoma (37). PTEN mutant mice were further analyzed for up to 2 years, and circulating estrogen increased the proliferation of thyroid follicular cells in the context of PI3K activation. At the same time, the expression of Tshr, Duox2, and Slc5a5 (sodium iodide symporter) in the thyroid of female mice was significantly higher, but not related to the PTEN state, indicating that estrogen has a vital function in controlling the expression of these genes. Complete estrogen loss through ovariectomy reduces the thyroid proliferative index of female mice to the same level as that of mutant males. As a result, circulating estrogen can lead to increased susceptibility to thyroid disease in women, at least a direct cause of activation of PI3K pathways (33). Estrogen-dependent proliferation in female mutants increases differential gene expression. PTEN -/- male mice have higher levels of Cdkn1b (p27) expression in the thyroid compared with female mice, which can be speculated that activation at least partly by controlling p27 levels leads to increased sensitivity of women to tumor transformation (33).

AKT is the core node of complex signal cascades and adjust PI3K through crosstalk and feedback loop. In PTC cells, AKT signals can be activated by E2 and bisphenol A (BPA) having similar structures with E2. In order to study the influence of low levels of E2 (0.1 mM−1 nM) on ER expression in PTC cell lines, protein levels, and mRMA levels of ERα, ERβ, and GPR30 (a kind of mER) were measured through RT-PCR assay, immunofluorescence assay, and Western imprinting. The effects of E2 and BPA are related to their concentration and time of action. E2 can enhance the expression of ERα/, ERβ, and GPR30 while rapidly phosphorylating AKT/mTOR. Furthermore, G-1 that is an agonist of G protein receptor 30 (GPR30) inhibits the expression of ERα but activates GPR30, while G-15 (GPR30 antagonists) reverses E2 effects on GPR30 and AKT/mTOR pathway (22). Moreover, blocking the upstream factors of AKT/mTOR pathway can significantly reduce the expression of signaling molecules of pathway outside the cell, so the rapid effect of E2 on PI3K/AKT/mTOR signaling pathways may require the interaction between mERα and GPR30. Abnormal stimulation of PI3K/AKT/mTOR pathways caused by estrogen may involve a variety of gene changes. Effects of estrogen on different molecules in PI3K/AKT/mTOR pathways should be further studied and new therapeutic targets to improve therapeutic efficacy for thyroid cancer patients should be found. In TC cells, RET-PTC can stimulate WNT/β-catenin pathways via phosphorylating β-catenin and activating the PI3/AKT pathway (39). E2 treatment leads to downregulation of β-catenin. The relationship between E2 and WNT/β-catenin pathways needs further exploration.

### MEK/ERK Pathway

Mutations or overexpression of upstream activators that activate MAPK signaling pathways are a common event in thyroid cancer (40). Estrogen stimulates thyroid tumor growth by MAPK cytoplasmic signals. E2 is a strong enhancing factor in thyroid tumor cells. E2 not only binds to the nuclear ER but also regulates the expression of mitogen-activated protein (MAP) kinase, and its activity is mainly regulated by thyroid cancer cell growth factor. E2 can make MAP kinase isozymes and ERK 1/2 highly phosphorylated in thyroid benign and malignant cells (27), and siERα or siERβ both can inhibit ERK phosphorylation induced by E2 (21). For example, in PTC cells (KAT5) and ATC cells (ARO), the imbalance between increased ERα and reduced ERβ enhances ERK1/2 activity, accompanied with the change in expression of apoptosis-related factor Bcl-2 and Bax, thereby leading to increased capacity for cell proliferation and survival (8). Furthermore, one study suggests that the MEK/ERK pathway is activated by E2 through a membrane-initiated non-genome signal pathway. In PTC BCPAP-ERα cells, E2 triggered rapid phosphorylation of ERK kinases, and E2-BSA as a kind of membrane that E2 cannot penetrate has also been discovered to rapidly increase the phosphorylation of ERK1/2. However, these effects are not found in ER-negative BCPAP cells. Cultured cells with U0126 (MEK/ERK pathway inhibitor) were used to verify whether the activation of the ERK1/2 pathway contributes to E2-mediated cell autophagy. U0126 significantly inhibited autophagy levels of PTC cells, consistent with previous studies that the MEK/ERK pathway can stimulate autophagy (28).

E2 growth stimulation of thyroid tumor cells is related to increased cyclinD1 expression. CyclinD1 regulate G1/S transformation of the cell cycle and cyclind1 genes have regulatory areas of estrogen response. Treatment of cells with E2 and MAP kinase 1 inhibitors PD098059 can prevent E2-induced cyclinD1 accumulation and estrogen-mediated mitosis, which in turn stimulates cell growth (27). ERα gene knock-down inhibited phosphorylated ERK1/2 and cyclinD1 expression levels (24). Moreover, ERα knockdown leads to IQ-domain GTPase-activating protein 1 (IQGAP1) overexpression, which has a negative effect on the association between expression of cyclinD1 and ERK pathway in FTC133 cell lines (24). The expression level of metastasis-associated lung adenocarcinoma transcript 1 (MALAT-1) and IQGAP1 are both increased in tissues of patients with thyroid cancer and thyroid cancer cells. MALAT-1 upregulated IQGAP1 expression, and knock-down IQGAP1 can rescue cell proliferation and migration of TC caused by MALAT-1 overexpression (41), which also triggered a significant decrease in ERα transcriptional activity (24). In co-transfected COS-7 cell lines and FTC-133 cell lines, its results showed that IQGAP1 was bound to ERα through co-immunoprecipitation. These results suggest that ERα, MALAT-1, and IQGAP1 participate in ERK pathways and regulate cyclinD1, Bcl-2, and Bax expression. ER-dependent DNA synthesis and transcription of cyclinD1 may be ERK-activated downstream factors induced by E2.

The development of ATC is related to abnormal action of the sodium iodide symporter (NIS). Endogenous NIS in thyroid cancer has adapted to the wide range of clinical applications of radioiodine therapy, which has been proven to be an effective way to eliminate malignant cells with minimal side effects for many years. Esrrg encodes estrogen-related receptor γ (ERRγ), and its reverse agonist enhances NIS-mediated uptake of radioactive iodine in ATC cells, thereby promoting reactivity to radioactive iodine therapy *in vitro* (34), followed with ERRγ protein downregulation and ERK 1/2 activation (26). Specific MEK inhibitors completely inhibit ERK1/2 activation and increased radioactive iodine uptake. Meanwhile ERRγ-regulated MAPK pathway performs a key role in regulating NIS function in ATC cells. ERRγ-specific reverse agonist GSK5182 increases the number of membrane localization NIS in ATC cells by activating the MAP kinase signal. Therefore, pre-exposure to GSK5182 enhances cytotoxic effect of ^131^I treatment (26). Proper regulation of these signaling pathways to enhance NIS function makes radioiodine therapy become a new therapeutic strategy for ATC. ERRγ reverse agonists can be used as adjuvants for ATC patients to improve their treatment effects.

### Reactive Oxygen Species-Related Pathways

In thyroid cell physiology, a great deal of reactive oxygen species (ROS) was generated by NADPH oxidase. This process is required by hormone biosynthesis but can cause high spontaneous gene mutation rates. The key function of ROS in activating autophagy has been proven early. Besides being an effective growth factor in TC, estrogen is also involved in other mechanisms about ROS production (42). That is to say, ERα participates in the development of thyroid cancer not only via enhancing proliferation of cells but also via a ROS-dependent manner to increase autophagy level that is an important survival-promoting catabolism process. The mitochondria are also a source of estrogen-responsive ROS in thyroid cancer cell lines, which is associated with UCP2 downregulation. Studies have shown that estrogen-related receptor α (ERRα) has significant impact for mitochondrial activity. Thyroid hormone increases ESRRA expression and activity by inducing PPARGC1A (a kind of transcriptional co-activator) through thyroid hormone receptor β1 (THRB1). Moreover, thyroid hormone can induce the activation of autophagy-regulated kinase ULK1 in an ESRRA-dependent manner (32).

Caroline C. Faria et al. (42) proposed a model of estrogen-induced in thyroid cells that increased ROS production. E2 can stimulate ROS production through self-metabolism or through NOX4 that may be located in the mitochondria, nuclear membrane, plasma membrane, or endoplasmic reticulum. ROS can enter the nucleus and produce a variety of changes that could lead to thyroid tumorigenesis. The estrogen metabolism pathway also induces DNA mutagenesis of desopurinine compounds (42). The level of intracellular ROS was measured by flow cytometry using an oxidative sensitive probe DCFH-DA. In ERα-positive BCPAP cell lines, E2 obviously improved the ROS level inside the cell but had less impact for normal BCPAP cells. Free radical scavengers NAC-inhibiting ROS can strongly attenuate PTC cell function and E2-induced autophagy. These researches indicate that E2 can increase ROS production via binding to ERα, thereby increasing cell autophagy levels in thyroid cancer (28).

## Estrogen-Mediated Signaling Pathways in the Tumor Microenvironment of Thyroid Cancer

Tumor microenvironment (TME) plays a more and more important role in understanding the complex biological mechanism of tumor. TME also affects the progress of TC. Immune cells and inflammatory cells in TME have important effects on the treatment and prognosis of TC (43). Early studies have proved that E2 promotes the development of many kinds of tumors mainly through the direct genomic and non-genomic effects on tumor cells. Nevertheless, the expression of ER, aromatic enzymes, and estrogen-related effects in the TME have shown that estrogen can also promote the progression of malignant tumors through immunosuppression (44), and other mechanisms, such as angiogenesis, hypoxia, and inflammation (45).

### VEGF Signal Pathway

Previous studies reported that VEGF expression has been found in multiple thyroid cancers. Estrogen increases angiogenesis in TC through regulating VEGF secretion of thyroid cells (46). In one study, estrogen could increase VEGF secretion by ER signal transduction to stimulate thyroid follicular carcinoma ML-1 cell lines, and then VEGF was applied to human umbilical vein endothelial cells (HUVECs), which enhanced microtubule production and migration. Further experiments demonstrated that estrogen-induced secreted paracrine factors of TC cells enhance endothelial cell production and metastasis. PI3K expression increases after supplementing E2 in thyroid cancer cells, followed by ICI addition, which decreased PIK3 expression. In addition, by adding neutralizing VEGF antibodies to the conditioned medium, the expression of PI3K decreased in all experimental groups, indicating that the expression of PI3K was mediated at least in part by VEGF signaling pathways (25). One study showed further that ERβ directly attenuated HIF-1α binding to the VEGF gene promoter through suppressing aryl hydrocarbon receptor nuclear translocator (ARNT) and then decreased the hypoxic induction of VEGF Mrna (47). The treatment about combining the use of anti-VEGF and anti-estrogen can inhibit ER and neutralize VEGF antibodies (25), and the study found that 3-3′-diindolylmethane (DIM) is a promising natural anti-estrogen drug that can be used as part of a treatment plan for TC (35). However, further experiments are required to verify the efficacy of these inhibitors at different concentrations and to elucidate the practical effects of combination therapy.

### HIF-1 Signal Pathway

Solid tumors are characterized by the rapid growth of tumor cells, and functionally effective vascular systems do not compensate them equally. Cancer cells and stromal cells need to overcome the differences in oxygen and nutrition levels caused by these, which leads to high heterogeneity in different regions of the tumor (48). Studies have shown that in all types of thyroid cancers, especially in FTC and ATC, the levels of HIF-1α were significantly higher than those in normal tissue (49). Estrogen contributes to the new vascularization of various tumors under hypoxia conditions, but the role of estrogen in the hypoxia environment of thyroid cancer is unclear. In human thyroid cancer cells, estrogen and hypoxia regulate HIF-1 signals, which can be inhibited by anti-estrogen-rich agents and YC-1 3-(5′-hydroxymethyl-2′-furyl)-1-benzylindazole (a kind of HIF-1 inhibitor). In addition, the effects of treating the medium of TC cells with estrogen leading to the migration of HUVECs and the production of microvascular vessels can be abolished by HIF-1 inhibitors (50).

### NF-κB Signal Pathway

Chronic inflammation is widely recognized as an auxiliary mechanism to promote tumor to progress. Molecular inflammatory processes play a central role in the malignant progression of TC, and NF-κB signaling plays an important role in tumor occurrence and inflammation-mediated. In the absence of leukocyte infiltration, the increase in NF-κB expression and activation in PTC is directly related, on the one hand, to the upregulation of proinflammatory genes (e.g., RAGE, P2X7R, COX2, NOS2, and MMP9) and, on the other hand, to the down-regulation of anti-inflammatory gene SOCS-1 (51). There are potential interactions between E2 pathways and modulators of tumor-promoting inflammation in breast and lung cancers (44). Genes associated with inflammation promote TC proliferation and activate various transcription factors in the presence of E2 (e.g., NF-κB and STAT family members). The role of ERα and ERβ in tumor-conditioned macrophages (TAM), IL-10, various inflammatory-related genes, and inflammatory cytokines, especially in the TME of thyroid cancer, needs further study (45).

## Cross-Talk Among Signal Pathways

The pathways related to the development of TC mostly act in combination, increasing the complexity of prognosis, progression, and pathogenesis on thyroid cancer (45). The genetic alteration belonging to the PI3K/AKT pathway promotes cell transformation from thyroid normal cells to FTC, while the genetic alteration of the MAPK pathway accelerates the transformation of cells to PTC. These two pathways can be activated by the accumulation of multiple mutations that promote the development of thyroid cells to aggressive TC and ATC (36, 38). While each of the RAS and PI3K signaling pathways alone cannot induce the transformation of follicular cells, their joint activation is significantly carcinogenic, thereby leading to aggressive thyroid follicular carcinoma. Furthermore, activating PI3K suppresses the feedback signal initiated by the KRAS, which in turn inhibits MAPK activity. Moreover, PI3K and KRAS synergistically, significantly upregulate mRNA levels of cyclinD1. So, co-inhibiting MAPK and PI3K pathways can entirely control malignant behavior of cell lines having double mutation of these two pathways, providing a strong reason to target these pathogenic pathways jointly in the treatment of thyroid cancer (37).

There may be crosstalk among E2, ROS, and VEGF in the development of TC. Estrogen in the thyroid gland can upregulate VEGF levels, and it has been shown that increased intracellular ROS in thyroid cancer causes HIF-1α overexpression, and sustained and stable release of VEGF, but antioxidants N-acetylcysteine can eliminate these effects (42). Moreover, increased intracellular ROS that is induced by E2 can positively regulate important oncogenic pathways, including the PI3K/AKT and ERK1/2 signaling pathways (42). Further studies demonstrate potential crosstalk among ROS, autophagy signaling, MAPK pathways, and ERα in PTC. The inhibition of ROS production by NAC weakens the activation of MAPK pathways triggered by EAPK in BCPAP-ERα cells, but the activation of the MAPK pathway is not affected by autophagy inhibitor CQ. Using U0126 to inhibit MEK activity does not affect E2-induced ROS production in BCPAP-ERα cell lines. To sum up, in PTC-ERα cells E2-mediated autophagy is related to MAPK pathway activation and ROS induction. At the same time, ROS production inside the cell can conduce pathway activation (28). Cadmium (Cd) is an effective metal estrogen, and its function is the same as G1 [ER and G protein-coupled estrogen receptor (GPER) agonist] and E2, inducing progression and metastasis in human TC cell lines (FRO and WRO). Cd can trigger ERK/AKT rapid activation, which then leads to NF-κB nuclear translocation, increasing the expression of cyclinD1 and cyclinA, and increasing the secretion of IL-8. Cd-induced cellular malignant phenotype can be inhibited by knockout GPER or some specific inhibitors of NF-κB, AKT, GPER, and ERK. These studies indicate that AKT/NF-κB and GPER/ERK pathways are associated with malignant behavior of TC cells that are GPER positive, which is induced by Cd (23). The crosstalk between ERα, HIF-1, and NF-α occurs in ER-α-positive TC, which can regulate gene expression in TC cells and promote pro-inflammatory and aggressive phenotypes (52).

## Estrogen Receptor-Mediated ceRNA Axis

E2 (1 nmol −1, 24 h) can promote the growth and maintenance of PTC stem cells, which induced dose-dependent cell proliferation and differentiation. *In vivo*, E2 promotes CSC mobility and tumorigenicity, and mice inoculated with thyroid cancer stem cells treated with estrogen have larger tumor blocks than mice in the control group (53). Further studies have shown that ERβ are significantly enhanced in papillary thyroid cancer stem cells (PTCSCs) and promote their ability to form spheres and tumor growth. Screening estrogen responsiveness to lncRNA in globular cell spheroid cells observed a significant increase in lncRNA-H19(H19) in PTCC and PTC tissue specimens (29). A retrospective, non-randomized study found that H19 is an independent risk factor for extrathyroid expansion and lymph node metastasis (54). ERβ can activate H19 transcription under E2 treatment, while ERβ silencing significantly inhibits stem-like characteristics of PTC cells induced by E2/H19. H19 knockdown rescues E2-mediated stem cell-like properties, and H19 releases ERβ expression sponged by miR-3126-5p in PTC cells (29). Other results have proven that miR-219-5p expression is negatively correlated with ERα expression. Importantly, ERα overexpression in PTC cells rescues the miR-219-5p inhibitory effects of cell migration and proliferation. The expression of miR-219-5p was decreased in PTC tissue samples. MiR-219-5p expression was statistically different in sex, tumor size, and lymph node metastasis of patients. Therefore, miR-3126-5p, by promoting ERβ, increases the growth of PTC cells, but miR-219-5p, by inhibiting ERα, plays an important role in the growth of PTC cells (31).

On treatment, it is first demonstrated that aspirin, a drug approved in the clinic, can significantly inhibit the differentiation and proliferation of PTC stem cells by reducing expression levels of lncRNAH19 and ERβ. Novel regulatory mechanisms of PTCSC can be regulated by aspirin, which may be potential therapeutic opportunities for patients (29). Shenmai injection (SMI) can enhance the antitumor effect of drugs and reduce the side effects of chemotherapeutic drugs. Treg cell ratio and the expression of Foxp3 in ^131^I radiotherapy patients were significantly increased compared with those who did not receive ^131^I treatment. SMI and ^131^I combined therapy reversed ^131^I-induced abnormal expression of GPER1 and miR-103 and inhibited CD4+T cell differentiation and reduced Treg cells, thereby improving postoperative immune function in ^131^I radiotherapy patients (30).

## Conclusion

Estrogen regulates different pathways through ERα and ERβ; ERα has a key role in tumorigenesis, and targeting its associated signaling pathways or ERβ can help us explore different therapeutic pathways. New treatments may include drugs that block ER activity by releasing ERβ and gene therapy. Studies have shown that low expression of ERβ is associated with poor prognosis and that all FTC patients who died of cancer-specific death had low ERβ expression (55). The presence of ERβ has a better prognosis and is associated with low-grade tumors, negative axillary lymph nodes, and higher disease-free survival (9). Therefore, ERβ can be used as a differential marker tool for preoperative malignant tumors, patients with low ERβ scores need more thorough follow-up and may benefit from more active treatment (55). However, one study has found no significant difference in ER-β mRNA levels in normal thyroid tissues compared with those in tumor tissue (56). The study also found lower levels of Erα mRNA in FTC and ATC, so estrogen is unlikely to play an important role in the progression of these cancers (56). At the same time, some studies have concluded that ERα expression is associated with tumors with good differentiation and reduces the incidence of disease recurrence (57), which is contrary to our previous conclusions.

Furthermore, exogenous expression of ERβ not only inhibits cell invasion of many cancer but also induces apoptosis of many cancer cells (9). Novel N-t-boc-hexylenediamine derivative of 7-(O)-carboxymethyl daidzein (cD-tboc) can mediate apoptosis and delay growth of human TC cells by inhibiting ERβ, which developed in the laboratory (58). Data from a prospective cohort study do not support the hypothesis that exogenous estrogen should be a causative factor or that estrogen deficiency is a protective factor for TC. For example, among women without hysterectomy or with hysterectomy plus bilateral salpingo-oophorectomy (BSO), the study did not find an obvious correlation between hormone therapy and thyroid cancer (59). Studies have also shown that the use of oral contraceptives after menopause is not associated with the risk of all thyroid cancers (60). However, for thyroid cancer patients, drugs containing estrogen or compounds that mimic estrogenic activity should be used carefully to prevent cancer progression, and should minimize exposure to endocrine disrupting chemicals (EDCs) that can produce estrogenic effects. At the same time, when evaluating the therapeutic effect and pathogenicity of ER antagonists or agonists, the interference of exogenous drugs should not be ignored.

Estrogen and estrogen receptor-mediated pathways are also related to other cancers: (1) Gynecologic cancers and breast cancer, (2) endocrine organ cancers, (3) digestive system cancers and lung cancer (61). For example, in lung cancer, E2 upregulated the expression of osteopontin (OPN), which contributes to the cross-talk between the ER and EGFR signaling pathways and estrogen-promoted cell migration through activating ERβ of the MEK/ERK signaling pathway (62). In endometrial carcinoma, there is an effect on the activation of the PI3K/AKT/mTOR transduction cascade via overexpression of ERα (63). Mechanisms and treatments in other cancers will provide a basis for further research on thyroid cancer.

Further exploration of the mechanism of estrogen in the progression of TC in the future can gradually uncover its magic. Understanding the key insights of the interactions of these pathways into how human cancer is transformed may guide the development of better treatment strategies for thyroid cancer.

## Author Contributions

JL: writing manuscripts and collecting literature. TX: collecting literature and modifying manuscripts. LM: collecting literature. WC: designing the content of the article and proposing amendments. All authors contributed to the article and approved the submitted version.

## Conflict of Interest

The authors declare that the research was conducted in the absence of any commercial or financial relationships that could be construed as a potential conflict of interest.
